# Safety and immunogenicity of inactivated COVID-19 vaccine in patients with metabolic syndrome: A cross-sectional observational study

**DOI:** 10.3389/fpubh.2022.1067342

**Published:** 2022-12-23

**Authors:** Qiao Guo, Lei Yang, Ran Peng, Tao Gao, Xinglin Chu, Depeng Jiang, Dazhi Ke, Hong Ren

**Affiliations:** ^1^Department of General Practice, The Second Affiliated Hospital, Chongqing Medical University, Chongqing, China; ^2^Department of Respiratory Medicine, The Second Affiliated Hospital, Chongqing Medical University, Chongqing, China; ^3^Key Laboratory of Molecular Biology for Infectious Diseases (Ministry of Education), Department of Infectious Diseases, Institute for Viral Hepatitis, The Second Affiliated Hospital, Chongqing Medical University, Chongqing, China

**Keywords:** inactivated COVID-19 vaccine, safety, immunogenicity, hypertension, hyperglycemia, hyperlipidemia

## Abstract

**Background and aims:**

The prevalence of metabolic syndrome (MS), wich mainly including hypertension, hyperglycemia, hyperlipidemia, remains high, and the safety and antibody response of inactivated coronavirus disease 2019 (COVID-19) vaccination in patients with metabolic syndrome (MS) is still inconsistency, therefore it is necessary to explore the safety and antibody responses of inactivated COVID-19 vaccination in MS patients in clinical practice.

**Methods:**

157 adults patients who were suffering from MS and 117 health controls (HC) at an interval of at least 21 days after full-course (2nd dose) vaccination were enrolled. The safety of inactivated COVID-19 vaccination was evaluated through collected adverse events (AEs) by questionnaire. The immunogenicity of included participant to inactivated COVID-19 vaccination was represented by serum seropositivity rate of anti-receptor binding domain (RBD) IgG, SARS-CoV-2 neutralizing antibodies (CoV-2 Nab) and titers of anti-RBD IgG, CoV-2 Nab. The B cells, mainly including RBD-specific B cells, RBD-specific memory B cell (MBC), RBD^+^ resting MBC cells, RBD^+^ activated MBC cells, RBD^+^ atypical MBC cells (atyMBCs), and RBD^+^ intermediate MBC cells, were also analyzed.

**Results:**

In terms of safety, all AEs in MS patients were mild and self-limiting, and the incidence was comparable to that of HC participants, with overall AEs within seven days reported in 9.6% (15/157) of 3H and 11.1% (13/117) of HC. Both groups experienced no serious adverse events. As for immunogenicity of MS patients to inactivated COVID-19 vaccination, compared with health controls, the seroprevalence of anti-RBD IgG and CoV-2 Nab was significantly decreased in MS patients (*p* = 0.000, *p* = 0.003, respectively), while the titers of anti-RBD IgG (AU/ml) and CoV-2 Nab (μg/ml) were also significant lower in MS patients (*p* = 0.014, *p* = 0.002, respectively). As for frequencies of B cells, MS patients had lower frequencies of RBD-specific B cells, RBD^+^ resting MBCs, and RBD^+^ intermediate MBCs (*p* = 0.003, *p* = 0.000, *p* = 0.000, respectively), but had a higher frequencies of RBD^+^ atypical MBCs (*p* = 0.000) than HC. In comorbidity number subgroups analysis of MS, except frequencies of RBD^+^ resting MBC cells, RBD^+^ activated MBC cells and RBD^+^ intermediate MBC cells had significant difference among three groups (*p* = 0.035, *p* = 0.042, *p* = 0.046, respectively), antibody response had no significant difference among 1H, 2H, and 3H groups (*p* > 0.05). And took 70 years old as a boundary, also no statistically significant differences (*p* > 0.05) were found in age subgroups. Lastly, comprehensive analysis in MS patients indicated that interval time after 2nd dose vaccine was the statistical significant factor which impacting antibody response in MS individuals.

**Conclusions:**

Inactivated COVID-19 vaccines were well-tolerated, but induced a poorer antibody response against SARS-CoV-2 in MS patients comparing to HC participants. Patients with MS should therefore be more proactive in receiving inactivated COVID-19 vaccine, and a booster vaccination may be considered necessary.

**Clinical trial registration:**

https://clinicaltrials.gov/, identifier: NCT05043246.

## Introduction

COVID-19, caused by SARS-CoV-2, which began in 2019 and spread globally, has emerged as a major global burden, posing serious public health challenges. As the current epidemic is still repeatedly and locally rising, vaccines should still be more widely accepted by the public as one of the effective preventive measures. However, the safety and effectiveness are always the main reasons why people are hesitant to get vaccinated (1). Patients with metabolic syndrome (MS), which mainly including hypertension, hyperglycemia, or hyperlipidemia (3H), have a greater risk of contracting the COVID-19 virus and a poorer prognosis than patients without those diseases (2–6). Due to the severity of the infection in patients with MS, prevention remains the mainstay. And wing to hypertension, hyperglycemia, hyperlipidemia complicating with immune dysfunction (4, 7–9), these MS individuals may benefit from COVID-19 vaccination. Although several inactived COVID-19 vaccines has been widely accepted currently, the safety and immunogenicity of inactived COVID-19 vaccines in MS patients is still inconsistency (10–12).

Previous studies has indicated that inactived COVID-19 vaccines were good tolerated in patients with hypertension, diabetes, and obesity or higher body mass index (BMI), but whether the immunogenicity existing difference between MS and individuals without MS is still inconsistency (10, 13–15). Watanabe et al. (10) reported that obesity and hypertension are associated with poorer antibody response to COVID-19 mRNA vaccine. Rifai et al. (13) found that vaccine effectiveness was declined in participants with hypertension, and the risk of being infected with COVID-19 was increased. Differently, Parthymou et al. (14) reported that compare to normol-weight individuals, the antibody response of overweight individuals had no significant difference. Besides, although subjects with diabetes and hypertension had lower antibody titres, this association was not statistically significant (14). Interestingly, Ali et al. (15) indicated that T2DM patients had lower anti-SARS-CoV-2 IgG and SARS-CoV-2 neutralizing antibodies (CoV-2 Nab) titers, but antibody titers did not change significantly with hypertension or obesity. It is obvious that immunogenicity of inactivated COVID-19 vaccine in MS population is still controversial, and clinicians lack corresponding evidence from clinical research to address MS patients' concerns.

In China and other nations across the world, inactivated vaccine is a common type of COVID-19 vaccination. This study sought to examine the safety and immune response of COVID-19 inactivated vaccines in MS individuals after a full course of immunization. Flow cytometry of B cells, particularly RBD-specific memory B cell (MBC), was detected to further investigate the mechanism of antibody response.

## Methods

### Participants

A total of 157 patients diagnosed with MS, mainly including hypertension, hyperglycemia, or hyperlipidemia and 117 healthy volunteers (HC) were continuously recruited from the Second Affiliated Hospital of Chongqing Medical University. Inclusion criteria for patients with hypertension, hyperglycemia, or hyperlipidemia, was made based on guideline (16–18). Hypertension diagnose main as following: (i) adults with an average SBP ≥140 mmHg or DBP ≥90 mmHg, (ii) adults were taking antihypertensive medication. Hyperglycemia main defined as meeting the diagnostic criteria of diabetes: (i) With typical symptoms, fasting blood glucose ≥7.0 mmol/L or postprandial blood glucose ≥11.1 mmol/L, (ii) No typical symptoms, only fasting blood glucose ≥7.0 mmol/L or postprandial blood glucose ≥11.1 mmol/L should be repeated once again, still reach the above value, (iii) No typical symptoms, only fasting blood glucose ≥7.0 mmol/L or postprandial blood glucose ≥11.1 mmol/L, glucose tolerance test 2 h blood glucose ≥11.1 mmol/L. Hyperlipidemia was determined based on present lipid levels or recent use of anti-dyslipidemic drugs. The key inclusion criteria for all individuals were: (i) after two dose vaccination [BBIBP-CorV (19) or Corona Vac (20)], (ii) the person of age ≥18 years. Important criteria for exclusion were: (i) prior COVID-19 infection, (ii) prior use of immune-suppressive medications within six months, (iii) auto-immune diseases, (iv) pregnancy, and (v) malignancy. This study has been registered at ClinicalTrials.gov (NCT05043246).

### Vaccines

Currently, the BBIBP-CorV and Corona Vac are the main components of vaccination in China. The Corona Vac is produced by Beijing Kexing Zhongwei Biotechnology Co., Ltd., and the BBIBP-CorV is produced by Sinopharmaceutical Group China Biology Beijing Biological products Research Institute Co., Ltd. (including Chengdu Institute, Lanzhou Institute and Changchun Institute), both are inactivated vaccines approved by the state.

### Data collection

Demographic and clinical data of MS and healthy controls were obtained by questionnaire and electronic medical record, and their peripheral blood was sampled at an interval of at least 21 days after the full-course vaccination for the detection of anti-RBD-specific IgG, CoV-2 Nab and RBD-specific B cells (Flow Chart at end of article). In the following analysis, participants were divided into two groups: MS group (*n* = 157), and health control group (HC) (*n* = 117). And for further analysis, MS patients were divided into three groups according to the number of combined MS diseases: 1H group (*n* = 68, patients with one of the MS), 2H group (*n* = 63, patients with two of the MS), and 3H group (*n* = 26, patients with all of the MS). And MS patients were also divided into two groups taking 70 years as a boundary: ≥70 years old group (*n* = 41) and <70 years old (*n* = 116).

### Safety and antibody response assessment

After full-course vaccination, all adverse events AEs within 7 days were classified in line with the scale published by the China Medical and Drug Administration (2019 edition). The overall incidence of adverse events within 7 days was compared between MS patients and healthy controls to assess safety. Titers of two antibodies, including anti-RBD IgG and CoV-2 Nab, after full-course vaccination were comprehensively compared and analyzed to assess antibody response.

### Adverse events monitoring

Participants' AEs were obtained by questionnaire and were verified by investigators. All AEs were graded according to the scale issued by National Medical Products Administration of China (version 2019), details information provided in supplementary material (Supplementary Tables 4A,B).

### Antibody responses testing

Capture chemiluminescence immunoassays were used to detect anti-RBD IgG and CoV-2 Nab in serum samples using MAGLUMI X8 (Snibe, Shenzhen, China) according to the manufacturer's instructions. The kits' (Snibe, Shenzhen, China) sensitivity and specificity for anti-RBD IgG were 100 and 99.6%, respectively, and both are 100% for NA bs. For anti-RBD IgG, the cut-off value was 1.00 AU/ml, and for NAbs, the cut-off value was 0.15 μg/ml.

### Detection of SARS-CoV-2 specific B cells by flow cytometry

The stained peripheral blood mononuclear cells were evaluated in the following procedure, using a Beckman flow cytometer (Beckman Coulter, Inc., California, USA). Antigen probe was obtained, and the biotinylated SARS-CoV-2 Spike RBD protein (40592-V08H2-B, Sino Biological, Beijing, China) was mixed with Streptavidin-BV421 (405225, Biolegend, California, USA) at a 4:1 molar ratio for 1 h. To obtain the peripheral blood mononuclear cells, density gradient centrifugation [Histopaque (10771, Sigma-Aldrich, St Louis, Missouri, USA)], cell cleaning (FACS, With 2% FBS), add antigen probes (1:33.3) and fluorescent-conjugated antibodies [anti-human IgG Fc (410722, Biolegend), anti-human IgM (314524, Biolegend) Antihuman CD3 (300430, Biolegend), anti-human CD19 (302212, Biolegend), anti-human CD21 (354918, Biolegend), anti-human CD27 (356406, Biolegend)] after being mixed and placed at 4°C, 30 min, conduct light-avoidance staining; Test sample (FACS buffer), Analyze the data [FlowJo software (V10.0.7)].

### Statistical analysis

To check the normality of data distribution the Shapiro-Wilk test was used. The data of normal distribution were presented as mean (SD), non-normal distribution as median (IQR). The differences between groups were compared, categorical variables were tested by chi-square test or Fisher exact and Student's *T*-test/Mann-Whitney U for continuous variables. Kruskal-Wallis test was used for continuous variables with three or more groups. Univariate and multivariate linear regression were performed to analyze the risk and protective factors of seropositivity rate of antibodies in MS individuals. All results are corrected. SPSS 26 (IBM Corp., Armonk, NY, USA) was used for data analysis and GraphPad Prism version 9.2.0 for drawing. We considered *P* < 0.05 to be statistically significant.

## Results

### Characteristics of the enrolled participants

As Table 1 showing, A total of 274 participants was included this study, which including 157 MS patients and 117 HC patients. The median age (62.00 vs. 61.00, *p* = 0.530), male (52.2 vs. 52.1%, *p* = 0.988), the mean body mass index (BMI) (24.45 vs. 23.34, *p* = 0.087), vaccine type (63.7 vs. 59.8%, *p* = 0.514), the median interval days after 2nd dose vaccination (42 vs. 54, *p* = 0.329), and blood routine (white blood cell, Hemoglobin, lymphocyte, Platelet) have no statistical differences between MS patients and HC. Additionally, though there have no significant statistical difference in BMI between MS patients and health controls, the BMI of MS participants is higher than health controls, which is consistent with the universal phenomenon.

**Table 1:** Characteristic of MS patients and health controls.

**Variables**	**MS Patients (*n* = 157)**	**Healthy controls (*n* = 117)**	***P*-value**
Age (years) median (Min, Max)	62 (19–89)	61 (19–87)	0.530
Gender [male, *n* (%)]	52.2% (82/157)	52.1% (61/117)	0.988
Body mass index^#^ (Kg/m^∧^2)	24.45 (16.60–48.83)	23.34 (16.65–33.57)	0.087
Vaccine type (Corona)	63.7% (100/157)	59.8% (70/117)	0.514
Days after 2nd dose vaccination median (IQR)	42 (16–168)	54 (20–142)	0.329
Red blood cell^#^ (10^∧^12/L)	4.49 (2.37–9.07)	4.57 (3.04–6.50)	0.067
White blood cell^#^ (10^∧^9/L)	6.15 (2.67–19.08)	5.97 (3.11–12.54)	0.077
Hemoglobin^#^ (10^∧^9 g/L)	137 (71–208)	138 (89–168)	0.405
Lymphocyte^#^ (10^∧^9/L)	1.68 (0.45–5.91)	1.71 (0.21–3.64)	0.396
Platelet^#^ (10^∧^9/L)	192 (80–1,182)	207 (100–638)	0.059

^#^Presented as median (Range). Chi-Square statistic test was used for categorical variables, and Mann-Whitney *U*-test was used for continuous variables. *P* < 0.05 was considered statistically significant.

### Safety assessment of inactived COVID-19 vaccination in MS patients

We found that the overall AEs rate (9.6 vs. 11.1%, *p* = 0.807), including local or systemic AEs were similar between MS patients and healthy controls, with no statistic difference. Besides, no severe adverse events (SAEs), which including grade 3 and 4, have occurred. In details, according to the questionnaire results, we found that the most common local and systemic AEs of MS patients were pain (4.5%, 7/157) and fatigue (3.2%, 5/157), respectively, and both are mild (grade 1 and 2) and self-limiting, which was similar with controls (details was shown in the Table 2).

**Table 2:** Adverse events of inactived COVID-19 vaccination in MS patients and health controls.

**Variable**	**MS patients (*n* = 157)**	**Healthy controls (*n* = 117)**	***P*-value**
Overall adverse events within 7 days	15 (9.6%)	13 (11.1%)	0.807
**Local adverse events**			
Pain	7 (4.5%)	7 (6.0%)	0.571
Swelling	1 (0.6%)	3 (2.6%)	0.420
Itch	2 (1.3%)	1 (0.9%)	1.000
Red	1 (0.6%)	1 (0.9%)	1.000
Numb	/	1 (0.9%)	1.000
**Systemic adverse events**			
Fatigue	5 (3.2%)	2 (1.7%)	0.705
Drowsiness	3 (1.9%)	4 (3.4%)	0.703
dizziness	1 (0.6%)	/	1.000
Fever	1 (0.6%)	1 (0.9%)	1.000
Cough	2 (1.3%)	1 (0.9%)	1.000
Epigastric pain	1 (0.6%)	/	1.000
Shoulder pain	/	1 (0.9%)	1.000
Gastro spasm	/	1 (0.9%)	1.000
Decreased hemoglobin	/	/	1.000
Decreased platelet count	/	/	1.000
Decreased albumin	/	/	1.000
Elevated liver enzymes	/	/	1.000
Grade 3 and 4 adverse events	/	/	1.000

Data are presented as *n* (%). Chi-square test and Fisher's exact test were used to compare statistical difference between groups. *P* < 0.05 was considered statistically significant.

### Antibody responses to inactived COVID-19 vaccination in MS patients

**Figure 1:**
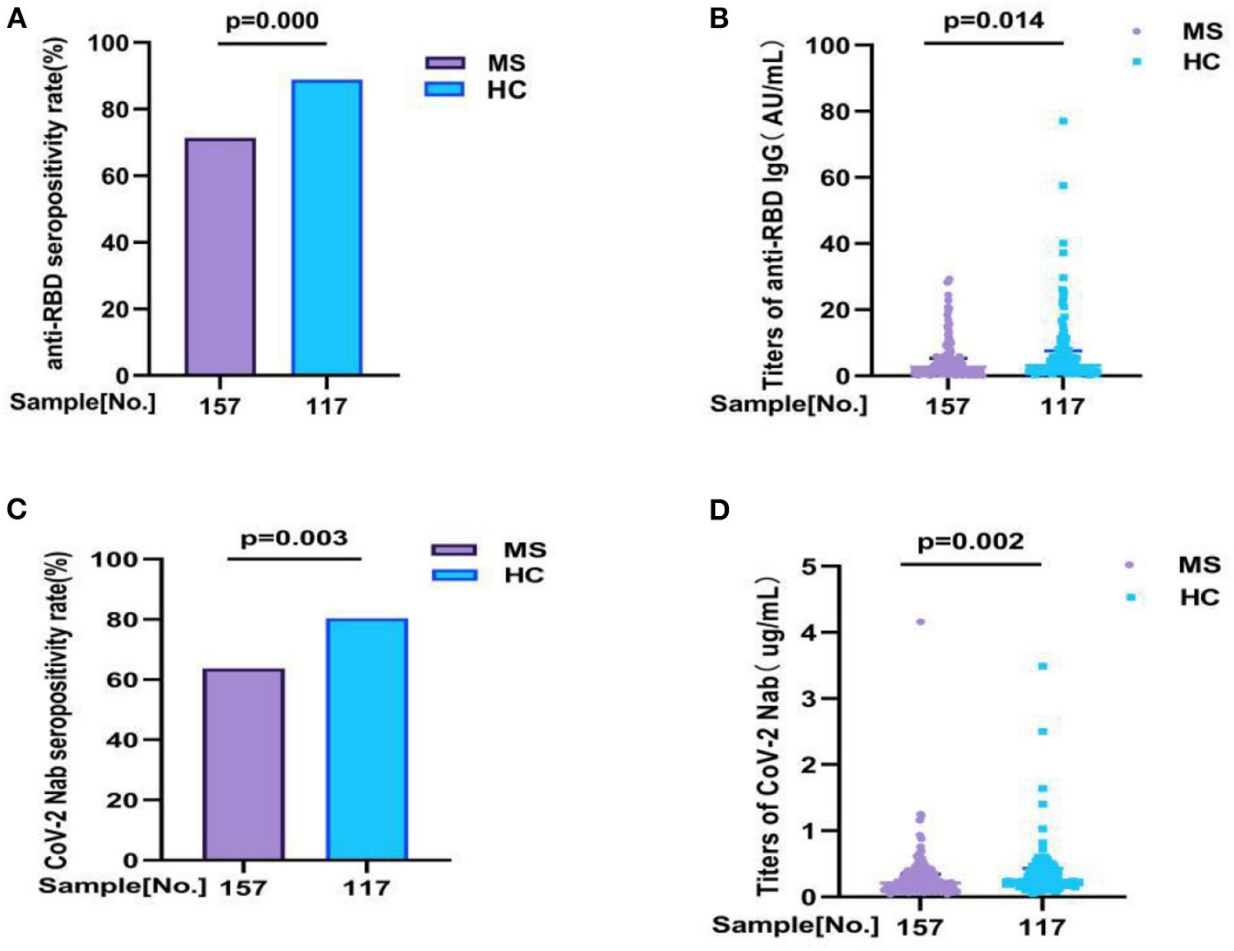
The antibody responses to inactivated COVID-19 vaccine in MS and HC. The antibody responses to inactivated COVID-19 vaccine in MS and HC. Overall, compared with HC, **(A,B)** the seropositivity rate of anti-RBD IgG and CoV-2 Nab was significant lower in MS patients (71.3 vs. 88.9%, *p* = 0.000, 63.70 vs. 80.34%, *p* = 0.003, respectively). Similarly, **(C,D)** the titers of anti-RBD IgG and CoV-2 Nab were also significant lower in MS patients: 2.650 (0.840, 5.300) vs. 3.150 (1.655, 7.550), *p* = 0.014; 0.210 (0.120, 0.345) vs. 0.250 (0.170, 0.430), *p* = 0.002, respectively. Titers were presented as median (IQR) (data are shown in Supplementary Table 1A).

In our study, we detected two antibodies for all participants (157 MS patients and 117 health controls) to evaluation the antibody response, including anti-RBD IgG and CoV-2 Nab. All participants were completed full-course (2nd dose) vaccination. Overall, compared with HC, the seropositivity rate of anti-RBD IgG and CoV-2 Nab was significant lower in MS patients (71.3 vs. 88.9%, *p* = 0.000, 63.70 vs. 80.34%, *p* = 0.003, respectively). Similarly, the titers of anti-RBD IgG (AU/ml) and CoV-2 Nab (μg/ml) were also significant lower in MS patients: 2.650 (0.840, 5.300) vs. 3.150 (1.655, 7.550), *p* = 0.014; 0.210 (0.120, 0.345) vs. 0.250 (0.170, 0.430), *p* = 0.002, respectively (Figure 1; Supplementary Table 1A).

### RBD-specific B cell responses to COVID-19 vaccinations

As the frequency and phenotype of B cells can showing humoral immune response to vaccine, the frequencies and phenotype of B cells, mainly including RBD-specific B cells, RBD-specific memory B cells (MBCs), RBD^+^ resting MBC, RBD^+^ activated MBCs, RBD^+^ atypical MBCs, RBD^+^ intermediate MBCs, were detected after full-course vaccination in all participants.

**Figure 2:**
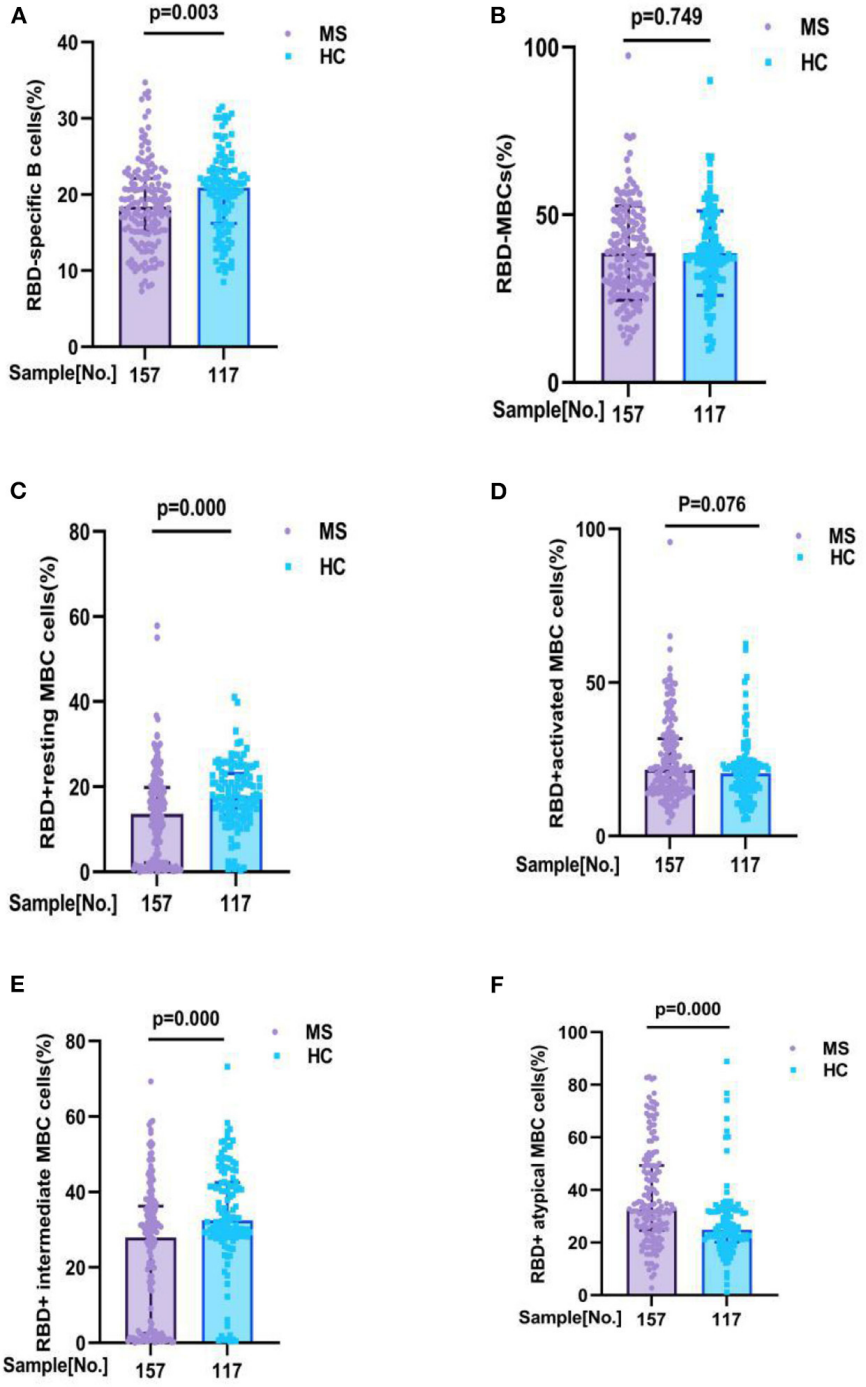
RBD-specific B cells response to inactivated COVID-19 vaccines in participants with MS and HC. RBD-specific B cells response to inactivated COVID-19 vaccines in participants with MS and HC. Comparisons of the RBD-specific B cells in all participants, MS patients had lower frequencies of **(A)** RBD - specific B cells (18. 42 v s. 20.90%, *p* = 0.003), **(C)** RBD+ resting MBCs (13.60 vs. 17.80%, *p* = 0.000), and **(E)** RBD+ intermediate MBCs (27.90 vs. 32.40%, *p* = 0.000) than HC, and had a higher frequencies of **(F)** RBD+ atypical MBCs (32.37 vs. 24.70%, *p* = 0.000) than HC. But no significant difference was found in the frequencies of **(B)** RBD-specific MBCs (37.80 vs. 37.70%, *p* = 0.749), **(D)** RBD+ activated MBCs (21.56 vs. 20.40%, *p* = 0.076), between MS patients and HC participants (data are shown in Supplementary Table 1B).

Comparisons of the RBD-specific B cells in all participants, we found that MS patients had lower frequencies of RBD-specific B cells (18.42 vs. 20.90%, *p* = 0.003), RBD^+^ resting MBCs (13.60 vs. 17.80%, *p* = 0.000), and RBD^+^ intermediate MBCs (27.90 vs. 32.40%, *p* = 0.000) than HC, and had a higher frequencies of RBD^+^ atypical MBCs (32.37 vs. 24.70%, *p* = 0.000) than HC. But no significant difference was found in the frequencies of RBD-specific MBCs (37.80 vs. 37.70%, *p* = 0.749), RBD^+^ activated MBCs (21.56 vs. 20.40%, *p* = 0.076), between MS patients and HC participants (Figure 2; Supplementary Table 1B).

### Antibody responses and RBD-specific B cell responses in 1H, 2H, 3H subgroups of MS

Additionally, to explore whether there is a significant difference in antibody response and RBD-specific B cell response among 1H, 2H, and 3H patients who were suffering from different number of MS, we divided MS patients into three subgroups (1H, 2H, 3H) and compared the antibodies response and RBD-specific B cells response to inactivated COVID-19 vaccines.

**Figure 3:**
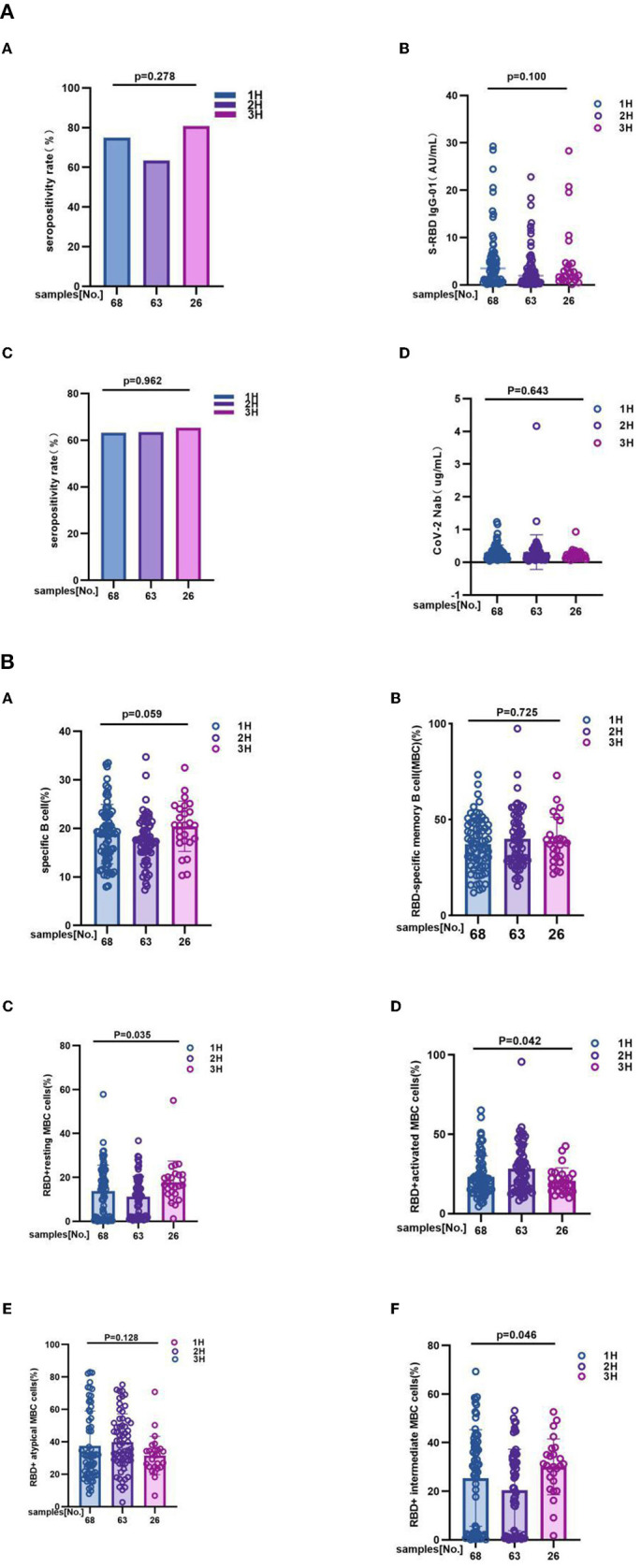
**(A)** The antibody responses to inactivated COVID-19 vaccines among 1H, 2H, and 3H subgroups of MS. The antibody responses to inactivated COVID-19 vaccines among 1H, 2H, and 3H subgroups of MS. It is different from overall analysis, for antibody response to inactivated COVID-19 vaccines had no significant difference among 1H, 2H, and 3H subgroups of MS. In details, (A) anti-RBD IgG seropositivity rate was 75.00 vs. 63.50% vs. 81.0%, *p* = 0.278, (B) titer of anti-RBD IgG was 3.450 (0.870, 5.910) vs. 1.950 (0.560, 4.430) vs. 2.235 (1.075, 4.595), *p* = 0.100; (C) CoV-2 Nab seropositivity rate was 63.2 vs. 63.5 vs. 65.4%, *p* = 0.962; (D) titer of CoV-2 Nab was 0.235 (0.120, 0.350) vs. 0.190 (0.110, 0.350) vs. 0.180 (0.120, 0.285), *p* = 0.643. titers were presented as median (IQR) (data are shown in Supplementary Table 2A). **(B)** The RBD-specific B cell responses to inactivated COVID-19 vaccines among 1H, 2H, and 3H subgroups of MS. 1H, 2H, and 3H represent patients who were suffering from different number of MS (Hypertension, Hyperglycemia, Hyperlipidemia). The results showing that frequencies of (C) RBD+ resting MBC cells (14.37 vs. 11.00 vs. 16.50%, *p* = 0.035), (D) RBD+ activated MBC cells (20.50 vs. 25.60 vs. 18.20%, *p* = 0.042), and (F) RBD+ intermediate MBC cells (29.34 vs. 21.30 vs. 30.50%, *p* = 0.046) had significant difference among three groups. But no significant difference was found in the frequencies of (A) RBD-specific B (19.05 vs. 17.50 vs. 20.67%, *p* = 0.059), (B) RBD-specific memory B cells (MBCs) (37.50 vs. 38.30 vs. 37.53%, *p* = 0.725), and (E) RBD+ atypical MBC cells (31.35 vs. 39.20 vs. 31.63%, *p* = 0.128) (data are shown in Supplementary Table 2B).

It is different from overall analysis, for antibody response to inactivated COVID-19 vaccines had no significant difference among 1H, 2H, and 3H subgroups. In details, anti-RBD IgG seropositivity rate was 75.00 vs. 63.50 vs. 81.0%, *p* = 0.278; titer of anti-RBD IgG was 3.450 (0.870, 5.910) vs. 1.950 (0.560, 4.430) vs. 2.235 (1.075, 4.595), *p* = 0.100; CoV-2 Nab seropositivity rate was 63.2 vs. 63.5 vs. 65.4%, *p* = 0.962; titer of CoV-2 Nab was 0.235 (0.120, 0.350) vs. 0.190 (0.110, 0.350) vs. 0.180 (0.120, 0.285), *p* = 0.643 (Figure 3A; Supplementary Table 2A). However, the results showing that frequencies of RBD^+^ resting MBC cells (14.37 vs. 11.00 vs. 16.50%, *p* = 0.035), RBD^+^ activated MBC cells (20.50 vs. 25.60 vs. 18.20%, *p* = 0.042), and RBD^+^ intermediate MBC cells (29.34 vs. 21.30 vs. 30.50%, *p* = 0.046) had significant difference among three groups. But no significant difference was found in the frequencies of RBD-specific B (19.05 vs. 17.50 vs. 20.67%, *p* = 0.059), RBD-specific memory B cells (MBCs) (37.50 vs. 38.30 vs. 37.53%, *p* = 0.725), and RBD^+^ atypical MBC cells (31.35 vs. 39.20 vs. 31.63%, *p* = 0.128) (Figure 3B; Supplementary Table 2B).

### Antibody responses and RBD-specific B cell responses in age subgroup

**Figure 4:**
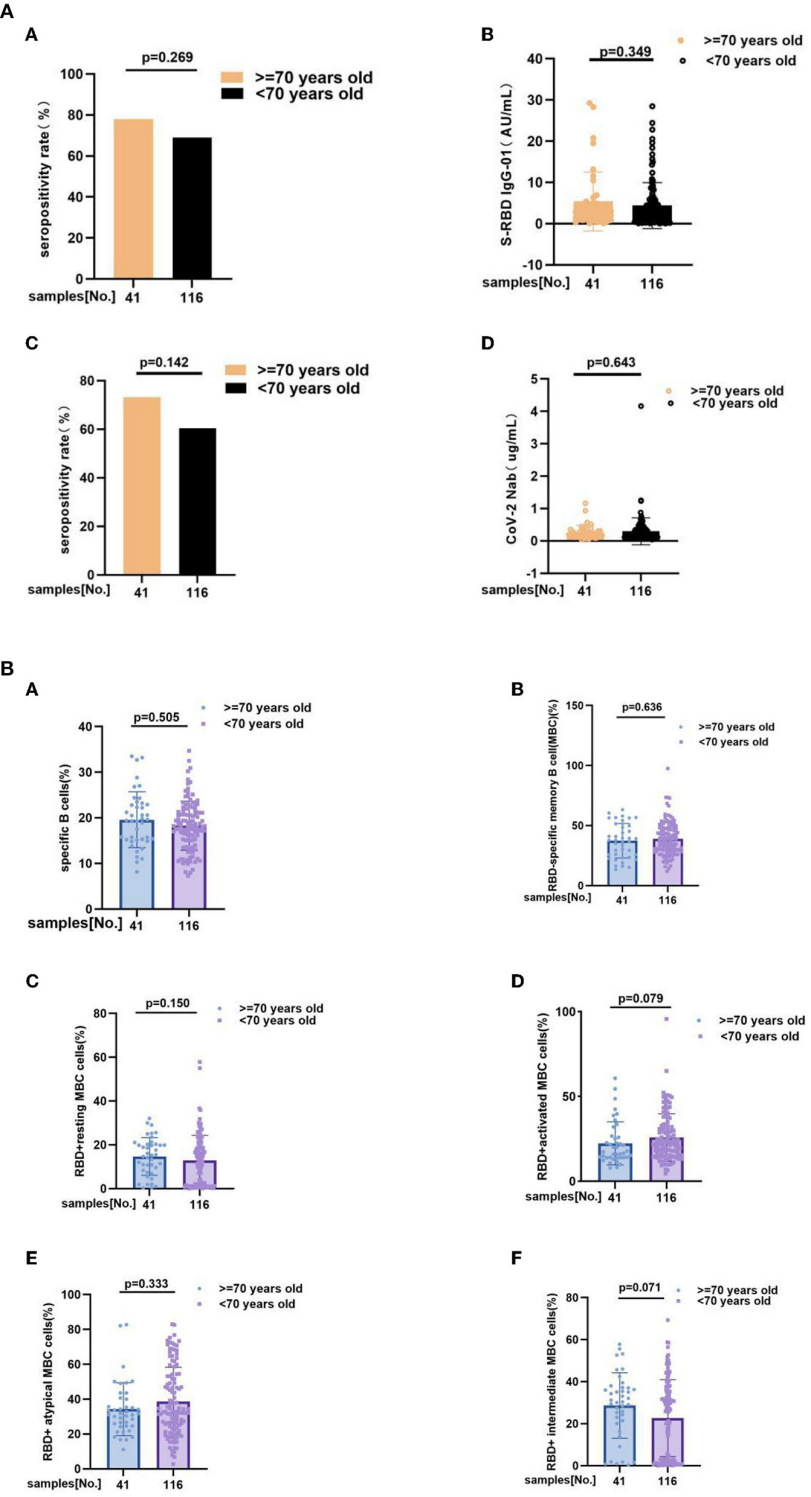
**(A)** The anti-RBD IgG and CoV-2 Nab responses to inactivated COVID-19 vaccines in ≥70 years old and <70 years old groups of MS. The anti-RBD IgG and CoV-2 Nab responses to inactivated COVID-19 vaccines in ≥70 years old and <70 years old groups of MS. Anti-RBD IgG seropositivity rate (A) (78 vs. 69%, *p* = 0.269), (B) titers of anti-RBD IgG as 2.780 (1.050, 4.955) vs. 2.445 (0.6625, 5.568), *p* = 0.349, (C) CoV-2 Nab seropositivity rate (73.2 vs. 60.3%, *p* = 0.142), (D) titers of CoV-2 Nab as 0.2200 (0.1300, 0.3050) vs. 0.1900 (0.1200, 0.3500), *p* = 0.643. Titers were presented as median (IQR). **(B)** RBD-specific B cells response to inactivated COVID-19 vaccines in MS patients at ≥70 years old and <70 years old. RBD-specific B cells response to inactivated COVID-19 vaccines in MS patients at ≥70 years old and <70 years old. Frequencies of (A) RBD-specific B cells (17.80 vs. 18.90%, *p* = 0.505), (B) RBD-specific memory B cells (MBCs) (37.80 vs. 37.78%, *p* = 0.636), (C) RBD+ resting MBC cells (15.20 vs. 12.76%, *p* = 0.150), (D) RBD+ activated MBC cells (17.30 vs. 22.30%, *p* = 0.079), (E) RBD+ atypical MBC cells (31.70 vs. 32.65%, *p* = 0.333), (F) RBD+ intermediate MBC cells (30.40 vs. 26.65%, *p* = 0.071) (the data are shown in Supplementary Tables 3A,B).

Take 70 years old as a boundary, we also individed MS patients into two subgroup, including ≥70 years old group (*n* = 41) and <70 years old group (*n* = 116). Subgroup analysis outcoming showed that neither of antibody responses and RBD-specific B cells response has significant difference between subgroups. Anti-RBD IgG seropositivity rate (78 vs. 69%, *p* = 0.269), titers of anti-RBD IgG as 2.780 (1.050, 4.955) vs. 2.445 (0.6625, 5.568), *p* = 0.349, CoV-2 Nab seropositivity rate (73.2 vs. 60.3%, *p* = 0.142), titers of CoV-2 Nab as 0.2200 (0.1300, 0.3050) vs. 0.1900 (0.1200, 0.3500), *p* = 0.643, frequencies of RBD-specific B cells (17.8 vs. 18.9%, *p* = 0.505), RBD-specific memory B cells (MBCs) (37.80 vs. 37.78%, *p* = 0.636), RBD^+^ resting MBC cells (15.20 vs. 12.76%, *p* = 0.150), RBD^+^ activated MBC cells (17.30 vs. 22.30%, *p* = 0.079), RBD^+^ atypical MBC cells (31.70 vs. 32.65%, *p* = 0.333), RBD^+^ intermediate MBC cells (30.40 vs. 26.65%, *p* = 0.071) (Figures 4A,B; Supplementary Tables 3A,B).

### Factors associated with lower antibody response to inactived COVID-19 vaccination in MS patients

On the bases of the resulting that both seropositivity rate and titers of anti-RBD-IgG and CoV-2 Nab in MS patients were lower than HC participants, we application univariate and multivariate analyses to further explore factors that affected this two antibodies. Through took demographic, clinical and immunological characteristics into univariate and multivariate ordinal logistic regression analyses, days after full-course (2nd dose) vaccination was negatively correlated with titers of anti-RBD IgG and CoV-2 Nab (OR = 0.968, *p* < 0.001; OR = 0.982, *p* = 0.002). At the same time, gender, BMI, vaccine type, combined number of MS, and even frequency of RBD-specific B cells were not significantly correlated with titers level of anti-RBD-IgG and CoV-2 Nab (*p* ≥ 0.05) in MS individuals (data are shown in Tables 3A,B).

**Table 3A:** Univariate and Multivariate analyses for anti-RBD-IgG in MS patients.

	**Univariate OR (95% CI)**	***P*-value**	**Multivariate OR (95% CI)**	***P*-value**
Gender (female)	0.643 (0.315, 1.291)	0.218	0.720 (0.303, 1.682)	0.450
Age (years)	1.016 (0.991, 1.041)	0.203	1.002 (0.971, 1.034)	0.894
BMI (Kg/m^∧^2)	1.009 (0.916, 1.111)	0.855	0.972 (0.868, 1.083)	0.595
Vaccine type	2.104 (1.036, 4.293)	0.039	2.149 (0.931, 5.024)	0.073
**Days after 2nd dose vaccine**	**0.973 (0.961, 0.985)**	**<0.001**	**0.968 (0.954, 0.981)**	**<0.001**
Number of combined MS	0.998 (0.620, 1.607)	0.993	1.088 (0.618, 1.950)	0.773
RBC (10^∧^12/L)	0.933 (0.554, 1.599)	0.792		
HB (10^∧^9 g/L)	1.004 (0.984, 1.023)	0.726		
WBC (10^∧^9/L)	0.949 (0.801, 1.132)	0.543		
LC (10^∧^9/L)	0.868 (0.510, 1.505)	0.597		
PLT (10^∧^9/L)	1.001 (0.997, 1.005)	0.764		
B cells/RBD-specific B cell (%)	1.012 (0.951, 1.079)	0.702	1.016 (0.943, 1.095)	0.677
B cells/RBD^+^ resting MBC cells (%)	1.027(0.993, 1.065)	0.138	5.479 (0.009, 7.430)	0.380
B cells/RBD^+^ activated MBC cells (%)	0.982 (0.958, 1.007)	0.150	4.024 (0.008, 4.169)	0.386
B cells/RBD^+^ atypical MBC cells (%)	0.992 (0.974, 1.010)	0.365	7.407 (0.011, 8.436)	0.343
B cells/RBD^+^ intermediate MBC cells (%)	1.012 (0.992, 1.033)	0.238	7.217 (0.011, 8.724)	0.344
B cells/ RBD-specific memory B cell (MBC) (%)	0.997 (0.973, 1.022)	0.802	1.550 (0.863, 2.720)	0.122

BMI, body mass index; MS, metabolic syndrome; RBC, red blood cell; WBC, white blood cell; HB, hemoglobin; LC, lymphocyte; PLT, platelet; RBD, receptor binding domain; MBC, memory B cell; CI, confidential interval; OR, odds ratio. *P* < 0.05 was statistically significant. The bold values means data has statistical differences.

**Table 3B:** Univariate and Multivariate analyses for CoV-2 Nab in MS patients.

	**Univariate OR (95% CI)**	***P*-value**	**Multivariate OR (95% CI)**	**P-value**
Gender (female)	0.699 (0.360, 1.342)	0.284	0.759 (0.357, 1.600)	0.469
Age (years)	1.011 (0.988, 1.034)	0.368	0.997 (0.970, 1.025)	0.840
BMI (Kg/m^∧^2)	1.050 (0.957, 1.163)	0.329	1.029 (0.932, 1.141)	0.572
Vaccine type	2.100 (1.073, 4.140)	**0.031**	1.973 (0.933, 4.198)	0.075
**Days after 2**^**nd**^ **dose Vaccine**	**0.985 (0.974, 0.995)**	**0.005**	**0.982 (0.969, 0.993)**	**0.002**
Number of combined MS diseases	1.040 (0.665, 1.639)	0.864	1.039 (0.626, 1.738)	0.884
RBC (10^∧^12/L)	0.908 (0.548, 1.499)	0.699		
HB (10^∧^9 g/L)	1.007 (0.988, 1.025)	0.492		
WBC (10^∧^9/L)	0.924 (0.780, 1.087)	0.336		
Lymphocyte (10^∧^9/L)	0.873 (0.521, 1.463)	0.596		
PLT (10^∧^9/L)	0.998 (0.994, 1.002)	0.284		
B cells/ RBD-specific B cell (%)	1.009 (0.951, 1.070)	0.775	1.003 (0.939, 1.072)	0.927
B cells/ RBD^+^ resting MBC cells (%)	1.040 (1.007,1.078)	0.023	3.244 (0.002, 6.710)	0.759
B cells/ RBD^+^ activated MBC cells (%)	0.982 (0.958, 1.005)	0.129	3.069 (0.002, 6.974)	0.771
B cells/ RBD^+^ atypical MBC cells (%)	0.985 (0.967, 1.002)	0.086	4.044 (0.002, 10.234)	0.722
B cells/ RBD+ intermediate MBC cells (%)	1.015 (0.997, 1.035)	0.108	4.037 (0.002, 10.097)	0.722
B cells/ RBD-specific memory B cell (MBC) (%)	1.004 (0.980, 1.027)	0.765	1.300 (0.770, 2.218)	0.315

BMI, body mass index; MS, metabolic syndrome; RBC, red blood cell; WBC, white blood cell; HB, hemoglobin; LC, lymphocyte; PLT, platelet; RBD, receptor binding domain; MBC, memory B cell; CI, confidential interval; OR, odds ratio.p < 0.05 was statistically significant. The bold values means data has statistical differences.

## Discussion

**Figure 5:**
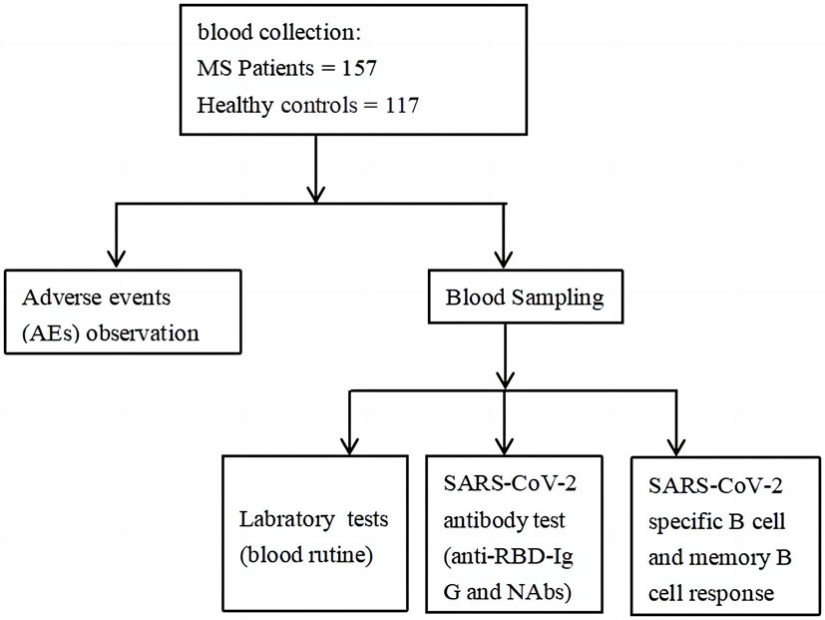
Flowchart of the study.

In this prospective study, we evaluated the safety by recording adverse events after full-course (2nd dose) inactivated SARS-CoV-2 (COVID-19) vaccines in MS individuals and HC participants (the flowchart as Figure 5). Meanwhile, the antibody responses and RBD-specific MBC responses were also assessed by FCM (flow cytometry).

Our findings demonstrated that inactivated COVID-19 vaccinations were safety in patients with MS, which is consistent with previous studies (10). The most common adverse events were local pain within 7 days of vaccination, with the incidence of 4.5% in MS patients. Similar results were seen in the healthy controls. No serious adverse events occurred in all participants, such as thromboembolic events (21), myocardial infarction (22) and so on, especially these older people with relatively low immunityt. Therefore, the safety of inactivated vaccine was further proved by this study.

It is critical to assess the effectiveness after full-course (2nd dose) vaccination to decide whether to give a booster dose. Therefore, after evaluating the safety of vaccination, we tested all the subjects for anti-RBD IgG and CoV-2 Nab. Our results showing that MS patients had a poorer antibody response than healthy controls, which mainly performed by antibody titers and seroprevalence. This is consistent with a series of previous studies (10, 13–15). In addition, through subgroup analysis based on the number of comorbid MS diseases, we discovered that there is no significant difference in antibody levels among the three subgroups. As a result, we hypothesized that the antibody response in this specific population would be similar, regardless of the number of comorbid MS diseases. MBCs are terminally differentiated cells that produce an innate immune response in response to antigen exposure. In the event of secondary infection, MBCs can proliferate and differentiate into antibody-secreting cells (ASCs), protecting the organism from disease (23, 24). Previous research has shown that RBD-specific B cells were good for antibody production (25). On the contrary, the frequencies of atyMBCs were found to be elevated in chronic inflammation, and several studies have reported that atyMBCs block the production of antibodies (26, 27). In this study, although no changes in the total numbers of MBCs could be seen, there was a decrease in the frequencies of RBD-specific B cells and a significant increase in the frequencies of atyMBCs in MS patients. Therefore, we speculate that humoral immunity may be weakening in this special population.

Multivariate linear regression analysis was performed in this study, we found that the interval after full-course (2nd dose) vaccination was associated with lower antibody levels, which is consistent with previous studies showing that antibody levels decrease over time (28, 29). Supplementary analysis based on vaccine type (details in the Supplementary Figure 6) revealed differences in antibody titers and positivity rates between the two vaccines (both the antibody titer and seropositivity rate induced by Corona Vac were higher than those induced by BBIBP-CorV), indicating that vaccine type is one of the factors influencing antibody response, consistent with the multivariate linear regression analysis results of vaccine type for antibody response and previous studies (30). Meanwhile, we also found that age was not associated with immune response in MS patients, but previous studies showed that age ≥70 was associated with a poorer antibody response in chronic obstructive pulmonary disease patients (COPD) (31) and the immune response was lower for persons over 55 years of age (32). These findings could be attributed to the small sample size, or they could indicate that the immune response would not have been significantly different in this specific population.

This cross-sectional study has a number of flaws. Firstly, the study was conducted in a single center with a small sample size. Secondly, no analysis of the participants' T cells was done in this study to compare the variations in immunogenicity following vaccination. Thirdly, no longitudinal analysis was done in this study, and only cross-sectional comparisons of pertinent antibody response indicators were done. Finally, the days after 2nd dose was unevenly matched because the sporadic localized outbreaks of COVID-19 partially impeded travel. Hence, subsequent patients will be enrolled one at a time and their antibodies and memory B cells will be examined. However, this study has some advantages. First, the safety and immunogenicity of the inactivated COVID-19 vaccine were studied in a MS population for the first time, which can help clinicians answer concerns that arise when MS patients receive vaccination. Second, this study provides a more thorough assessment of the vaccine's safety, antibody response, and RBD-specific B cells response. Third, clinical data confirmed the time interval following full vaccination as a risk factor affecting anti-RBD-IgG and CoV-2 Nab levels.

In conclusion, antibody responses to inactivated COVID-19 vaccines are reduced in MS patients, and antibody levels decline over time after vaccination, but this patient population has a high safety profile and tolerates COVID-19 inactivated vaccine well. Patients with MS should therefore be more proactive in receiving inactivated COVID-19 vaccine, and a booster vaccination may be considered necessary.

## Data availability statement

The original contributions presented in the study are included in the article/Supplementary material, further inquiries can be directed to the corresponding author/s.

## Ethics statement

The studies involving human participants were reviewed and approved by the Second Affiliated Hospital of Chongqing Medical University's Ethics Committee. The patients/participants provided their written informed consent to participate in this study.

## Author contributions

QG, LY, DJ, DK, and HR: conceptualization and methodology. DJ, DK, and HR: project administration. QG, LY, RP, TG, and XC: data collection and questionnaire. QG and LY: software, graphing, and writing. All authors contributed to the article and approved the submitted version.
