# Supplementary material for: Safety and immunogenicity of inactivated COVID-19 vaccine in patients with metabolic syndrome: A cross-sectional observational study
Source: Front Public Health. 2022 Dec 23;10:1067342. doi: 10.3389/fpubh.2022.1067342 (PMC9817001; doi:10.3389/fpubh.2022.1067342)
Supplement: Supplementary file 1 [file Data_Sheet_1.pdf]

Figure 6: Analysis of the differences between the two vaccines in terms of antibody response and RBD-specific B cell response.

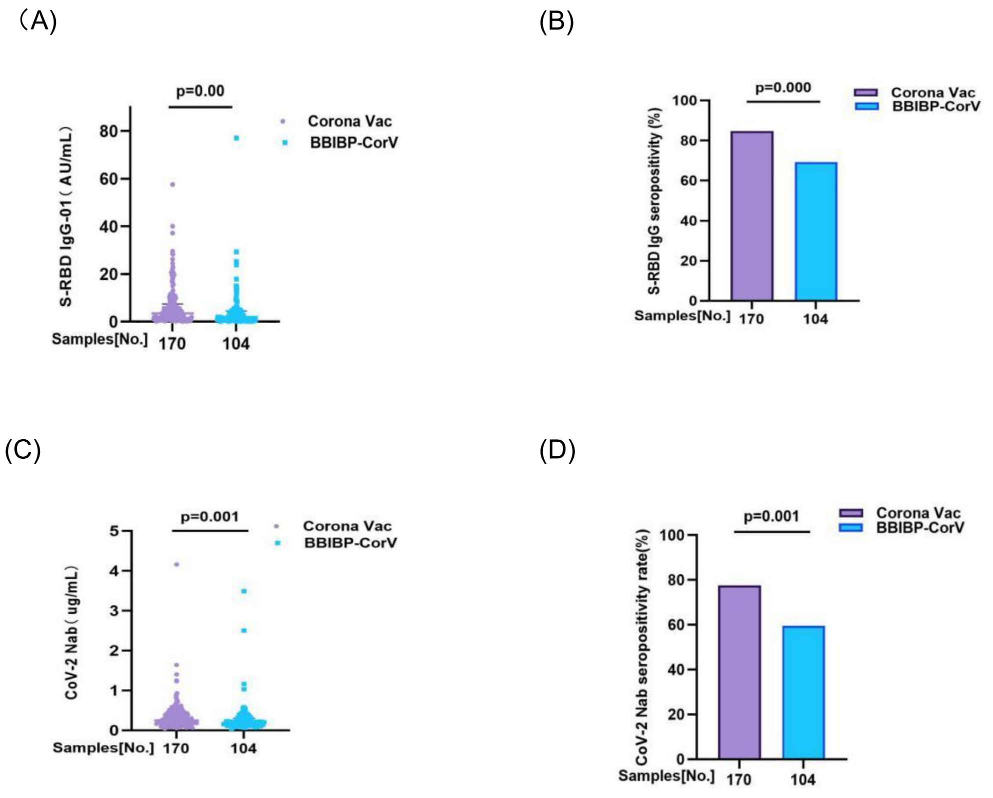

As a result, both the antibody titers and seropositivity rate induced by Corona Vac were higher than those of BBIBP-CorV, while there is no difference in RBD-specific B cell response: titer of anti-RBD IgG was 3.525 (1.713,7.435) *vs.* 1.955 (0.860,4.438), p = 0.000, anti-RBD IgG seropositivity rate was 84.71% *vs.* 69.23%, p = 0.002; titer of CoV-2 Nab was 0.250 (0.160,0.430) *vs.* 0.190 (0.123,0.300), p = 0.001, CoV-2 Nab seropositivity rate was 77.65% *vs.* 59.61%, p = 0.000 (**Figure6 (A)-(D)).**

(E)

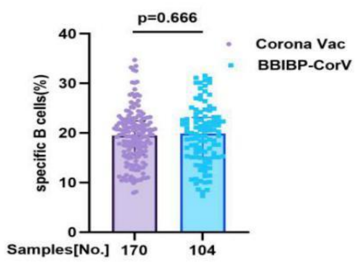

(F)

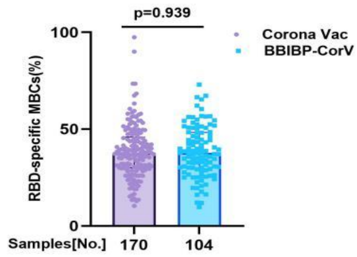

(G)

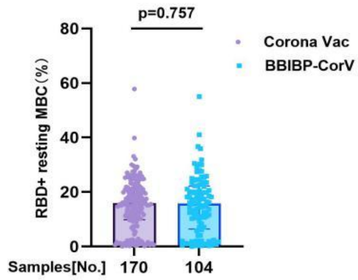

(H)

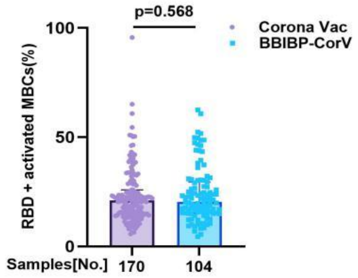

(I)

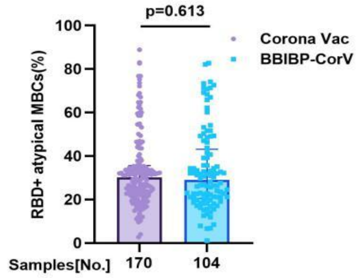

(J)

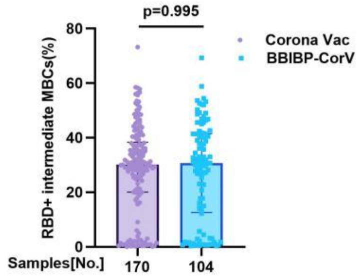

Frequencies of specific B cells (%): 19.50(15.63,22.41)vs. 19.90(15.10,23.15), p=0.666; frequencies of RBD-specific MBCs (%): 37.77(30.05,45.98)vs. 37.70(29.63,48.35), p=0.939; frequencies of RBD+ resting MBCs (%): 15.98(9.825,21.23)vs. 15.75(6.405,22.23), p=0.757; frequencies of RBD+ activited MBCs (%): 21.08(14.80,25.93)vs. 20.45(15.30,29.50), p=0.568); frequencies of RBD+ atypical (%): 30.21(22.60,35.63)vs. 29.00(20.53,43.15), p=0.613; frequencies of RBD+ intermediate MBCs (%): 30.13(20.08,38.38)vs. 30.71(12.60,41.40), p=0.995. (**Figure 6 (E)-(J))**.
