# Supplementary material for: Safety and immunogenicity of inactivated COVID-19 vaccine in patients with metabolic syndrome: A cross-sectional observational study
Source: Front Public Health. 2022 Dec 23;10:1067342. doi: 10.3389/fpubh.2022.1067342 (PMC9817001; doi:10.3389/fpubh.2022.1067342)
Supplement: Supplementary file 2 [file Data_Sheet_2.pdf]

Supplementary Material

Supplementary Tables

Supplementary Table1A (seropositivity rates and titers of anti-RBD and CoV-2 Nab in all participants)

	3H patients (n = 157)	Health controls (n = 117)	p vaule
anti-RBD seropositivity rate (%)	71.30	88.90	**0.000**
Titers of anti-RBD IgG （AU/ml）	2.650 (0.840,5.300)	3.150 (1.655,7.550)	**0.014**
CoV-2 Nab seropositivity rate (%)	63.70	80.34	**0.003**
Titers of CoV-2 Nab（μg/ml）	0.210 (0.120,0.345)	0.250 (0.170,0.430)	**0.002**

$p < 0.05$ was statistically significant. Titers were presented as median (IQR).

Supplementary Table1B (B cells flow cytometry in all participants)

	3H group (n = 157)	HC group (n = 117)	p value
RBD-specific B cells (%)	**18.42 (15.10,22.10)**	**20.90 (16.20,23.25)**	**0.003**
RBD-specific memory B cells (MBCs) (%)	37.80 (28.60,48.20)	37.70 (31.65,44.60)	0.749

RBD$^+$ resting MBC cells (%)	**13.60 (2.10,19.9)**	**17.80 (13.21,23.10)**	**0.000**
RBD$^+$ activated MBC cells (%)	21.56 (14.90,31.75)	20.40 (15.15,23.62)	0.076
RBD$^+$ atypical MBC cells (%)	**32.37 (24.55,49.25)**	**24.70 (20.10,32.21)**	**0.000**
RBD$^+$ intermediate MBC cells (%)	**27.90 (2.50,36.20)**	**32.40 (28.14,42.50)**	**0.000**

$p < 0.05$ was statistically significant.Titers were presented as median (IQR).

Supplementary Table 2A (antibody response in 1H, 2H, 3H)

	1H (n = 68)	2H (n = 63)	3H (n = 26)	p value
S-RBD seropositivity rate (%)	75.00	63.50	81.0	0.278
S-RBD IgG-01（AU/ml）	3.450 (0.870,5.910)	1.950 (0.560,4.430)	2.235 (1.075,4.595)	0.100
CoV-2 Nab seropositivity rate (%)	63.2	63.5	65.4	0.962
CoV-2 Nab（μg/ml）	0.235 (0.120,0.350)	0.190 (0.110,0.350)	0.180 (0.120,0.285)	0.643

$p < 0.05$ was statistically significant.Titers were presented as median (IQR).

Supplementary Table 2B (RBD-specific B cell responses in 1H, 2H, 3H)

	1H (n = 68)	2H (n = 63)	3H (n = 26)	p value

RBD-specific B cells (%)	19.05(14.20,22.75)	17.50(15.10,21.10)	20.67(17.34,24.18)	0.059
RBD-specific memory B cells (MBCs) (%)	37.50(25.93,47.98)	38.30(28.80,50.80)	37.53(29.65,44.03)	0.725
RBD^{+}resting MBC cells (%)	**14.37(1.188,22.53)**	**11.00(1.870,18.20)**	**16.50(12.48,21.15)**	**0.035**
RBD^{+}activated MBC cells (%)	**20.50(14.83,28.95)**	**25.60(15.80,38.10)**	**18.20(13.98,24.88)**	**0.042**
RBD^{+} atypical MBC cells (%)	31.35(20.53,54.18)	39.20(28.40,51.60)	31.63(24.50,35.18)	0.128
RBD^{+} intermediate MBC cells (%)	**29.34(2.438,39.88)**	**21.30(0.950,33.90)**	**30.50(23.35,35.75)**	**0.046**

$p < 0.05$ was statistically significant.

Supplementary Table 3A (antibody response in participants at ≥ 70 years old and <70 years old)

	≥ 70 years old (n = 41)	< 70 years old (n = 116)	p value
anti-RBD IgG seropositivity rate (%)	78.0	69.0	0.269
anti-RBD IgG （AU/ml）	2.780 (1.050,4.955)	2.445 (0.6625,5.568)	0.349
CoV-2 Nab seropositivity rate (%)	73.2	60.3	0.142
CoV-2 Nab （µg/ml）	0.2200	0.1900	0.643

	(0.1300,0.3050)	(0.1200,0.3500)	

$p < 0.05$ was statistically significant. Titers were presented as median (IQR).

Supplementary Table 3B (RBD-specific B cell responses in participants at ≥ 70 years old and < 70 years old)

	≥ 70 years old (n = 41)	< 70 years old (n = 116)	p value
RBD-specific B cells (%)	17.80 (14.95,21.10)	18.90 (15.13,22.28)	0.505
RBD-specific memory B cells (MBCs) (%)	37.80 (24.65,50.65)	37.78 (29.30,48.03)	0.636
RBD^+ resting MBC cells (%)	15.20 (7.890,20.25)	12.76 (1.630,19.25)	0.150
RBD^+ activated MBC cells (%)	17.30 (14.00,26.30)	22.30 (15.23,32.40)	0.079
RBD^+ atypical MBC cells (%)	31.70 (24.60,40.35)	32.65 (23.75,53.20)	0.333
RBD^+ intermediate MBC cells(%)	30.40 (20.75,37.10)	26.65 (1.985,35.70)	0.071

$p < 0.05$ was statistically significant.

Supplementary Table 4A.

Grading of adverse reactions:

The grading of systemic and local and other adverse reactions after vaccination refers to the State Food and Drug Administration's Guidelines for Grading Criteria for Clinical Observation of Adverse Reactions to Vaccines for Prophylaxis and the evaluation criteria of the National Institute of Allergy and Infectious Diseases (NIAID) of the National Institutes of Health Observatory (NIH), see the Grading Scale for Side Effects and Toxicity in Children and Infants. as shown below.

Supplementary table 4A Local reaction grading table

Local reaction	Mild (Grade1)	Moderate (Grade 2)	Severe (Grade 3)	Potentially life-threatening (Grade 4)
Pain	Does not interfere with activities	Interferes with activities or repeated use of non-narcotic pain medication	Interferes with daily activities or repeated use of narcotic pain medication	Emergency or hospitalization
Skin Mucosa	Red, itchy	Diffuse, maculopapular rash,rash, dry, flaky	Vesicular, moist, flaky or ulcerated	Desquamative dermatitis involving mucous membranes, or erythema multiforme, or suspected Stevens-Johnsons syndrome
Hard knot*	< 15 mm	15~30 mm	> 30 mm	Gangrenous or exfoliative dermatitis
Red*	< 15mm	15~30 mm	> 30 mm	Gangrene or exfoliative dermatitis
Swelling**	< 15mm and does not affect activity	15-30 mm or interferes with activity	> 30 mm or restricts daily activities	Gangrene

Frequently active rash (injection part)	< 15mm	15~30 mm	> 30 mm	
Pruritus	Injection site slightly	Injection limb moderately itchy	Itchy all over the body	

*In addition to the most direct grading of the local response by measuring the diameter, the developmental changes in measurements should be recorded; **evaluation and grading of swelling should be based on functional class and actual measurements

Supplementary table 4B Systemic reaction grading scale

Systemic reaction	Mild (Grade 1)	Moderate (Grade 2)	Severe (Grade 3)	Potentially life threatening (Grade 4)
Fever (axillary temperature)	37.1~37.5°C	37.6~39.0°C	> 39.0°C	
Allergic reactions	Pruritus without rash	Localized urticaria	Generalized urticaria, angioedema	Severe allergic reactions
Tiredness and	Slight mental	Slightly	Mental	Medical or

fatigue	depression, reduced mobility <48 hours	depressed, depressed, 20%-50% reduction in activity	depression, weakness, > 50% reduction in activity, or > 72 hours	hospitalization required
Vomiting	1 time per day, intake basically normal and does not affect activity	2~3 times a day, significant decrease in intake, or limited activity	4-6 times a day, no significant intake, intravenous fluids required	More than 6 times a day or severe vomiting
Diarrhea	Mild or transient. 2~3 loose stools/day, or mild diarrhea lasting less than 1 week	Moderate or persistent, 4-5 times/day, or diarrhea > 1 week	> 6 watery stools/day, or bloody diarrhea, upright hypotension, electrolyte imbalance, requiring intravenous fluids > 2L	Hypotensive shock requiring hospitalization
Convulsions	-	1~2 times, no medication required	More than 2 times, medication required	Shock, requiring hospitalization
Crying	Slightly more than usual	1 time more than usual	More than 1 times more than usual and lasts more than 3 days	Need to be seen and hospitalized
Lactation and eating disorder	Slight decrease in the number	The number of times or	The number of meals or the	Need to be seen and

	or amount of food eaten compared to usual	amount of food eaten is 1 times less than usual	amount of food eaten is more than 1 times less than usual and lasts for more than 1 day	hospitalized
Cough	Transient, no treatment needed	Persistent cough, treatment effective	Paroxysmal cough, uncontrolled by treatment	Emergency or hospitalization

For clinical abnormalities not covered in the grading scale above, the intensity of the adverse reactions was assessed according to the following criteria:

Grade 1 Mild Short duration of discomfort (<48 hours), no medical treatment required;

Grade 2 Moderate Mild to moderate restriction of daily activities, requiring no or minimal medical intervention;

Grade 3 Severe Significant restriction of daily activities, requiring daily care, medical treatment and possible hospitalization;

Grade 4 life-threatening Extremely limited in daily activities, significant need for daily care, medical treatment and hospitalization.
